# Testing Suicide Risk Prediction Algorithms Using Phone Measurements With Patients in Acute Mental Health Settings: Feasibility Study

**DOI:** 10.2196/15901

**Published:** 2020-06-26

**Authors:** Alina Haines-Delmont, Gurdit Chahal, Ashley Jane Bruen, Abbie Wall, Christina Tara Khan, Ramesh Sadashiv, David Fearnley

**Affiliations:** 1 Faculty of Health, Psychology and Social Care Manchester Metropolitan University Manchester United Kingdom; 2 CLARA Labs CLARA Analytics Santa Clara, CA United States; 3 University of Liverpool Health Services Research Liverpool United Kingdom; 4 Stanford University School of Medicine Stanford, CA United States; 5 Mersey Care NHS Foundation Trust Prescot United Kingdom

**Keywords:** suicide, suicidal ideation, smartphone, cell phone, machine learning, nearest neighbor algorithm, digital phenotyping

## Abstract

**Background:**

Digital phenotyping and machine learning are currently being used to augment or even replace traditional analytic procedures in many domains, including health care. Given the heavy reliance on smartphones and mobile devices around the world, this readily available source of data is an important and highly underutilized source that has the potential to improve mental health risk prediction and prevention and advance mental health globally.

**Objective:**

This study aimed to apply machine learning in an acute mental health setting for suicide risk prediction. This study uses a nascent approach, adding to existing knowledge by using data collected through a smartphone in place of clinical data, which have typically been collected from health care records.

**Methods:**

We created a smartphone app called Strength Within Me, which was linked to Fitbit, Apple Health kit, and Facebook, to collect salient clinical information such as sleep behavior and mood, step frequency and count, and engagement patterns with the phone from a cohort of inpatients with acute mental health (n=66). In addition, clinical research interviews were used to assess mood, sleep, and suicide risk. Multiple machine learning algorithms were tested to determine the best fit.

**Results:**

K-nearest neighbors (KNN; k=2) with uniform weighting and the Euclidean distance metric emerged as the most promising algorithm, with 68% mean accuracy (averaged over 10,000 simulations of splitting the training and testing data via 10-fold cross-validation) and an average area under the curve of 0.65. We applied a combined 5×2 *F* test to test the model performance of KNN against the baseline classifier that guesses training majority, random forest, support vector machine and logistic regression, and achieved *F* statistics of 10.7 (*P*=.009) and 17.6 (*P*=.003) for training majority and random forest, respectively, rejecting the null of performance being the same. Therefore, we have taken the first steps in prototyping a system that could continuously and accurately assess the risk of suicide via mobile devices.

**Conclusions:**

Predicting for suicidality is an underaddressed area of research to which this paper makes a useful contribution. This is part of the first generation of studies to suggest that it is feasible to utilize smartphone-generated user input and passive sensor data to generate a risk algorithm among inpatients at suicide risk. The model reveals fair concordance between phone-derived and research-generated clinical data, and with iterative development, it has the potential for accurate discriminant risk prediction. However, although full automation and independence of clinical judgment or input would be a worthy development for those individuals who are less likely to access specialist mental health services, and for providing a timely response in a crisis situation, the ethical and legal implications of such advances in the field of psychiatry need to be acknowledged.

## Introduction

### Background

Limitations in scalability, accuracy, and consistency with respect to traditional methods of predicting suicidal behavior have been recognized in the literature and meta-analyses [1-5]. Suicidality has been defined as any suicide-related behavior, including completing or attempting suicide (intent), suicidal ideation (thoughts), or communications [6]. Not everyone who experiences suicidal ideation attempts suicide, but suicidal thoughts have been shown to be linked to a higher risk of death by suicide [7]. Although some people communicate their suicidal thoughts or plans to friends and family before suicide, others do not disclose their intent [8-10]. In addition, some individuals might not seek help during a time of crisis because of various perceived constraints, including fear of stigma or disclosure, lack of time, access to services, and preference for informal help [11]. Our ability to predict suicide is limited by our understanding of suicidal thoughts and their nature [12].

### Suicidal Ideation, Smartphone apps, and Machine Learning

Advances in smartphones and connected sensors (wearables) have opened new possibilities for real-time, context-related monitoring of suicidal thoughts and suicidal risk [13], for example, ecological momentary assessments [14] that allow self-reporting of suicidal thoughts as they occur in an individual’s day-to-day life, naturalistic setting [15] and digital phenotyping that enables access to real-time classification and quantification of human behavior [16-18].

The use of computational data-driven methodologies that use social media to understand health-related issues (infodemiology, infoveillance [19,20]) and data mining techniques (artificial intelligence, machine learning algorithms [21]) provides additional potential in expanding our understanding of people’s thoughts, feelings, behavior, etc and improving monitoring of suicide risk in real time. Although in its infancy, new research exploring suicidal ideation has shown that social media (eg, Twitter and Facebook) could potentially be used as a suicide prevention tool [10,22-26]. One study, for example, demonstrated the utility of social media blog post analysis in classifying individuals with high suicide risk in China [27]. Some research indicates that by analyzing certain patterns of smartphone use, changes in mental health symptoms could be identified [28].

Although standardized clinical tools can help to classify factors that contribute to suicide risk and understand biological markers related to suicide (trait analyses), computer science and machine learning can provide additional and timely tools to understand linguistic markers of suicide thought (state analyses) [29]. New statistical methods have been proposed and tested to achieve more accurate predictions of risk, for example, support vector machines (SVMs), deep neural nets, and random forests [13]. Evidence suggests that these methods, especially elastic net, perform better than traditional logistic regression techniques [30]. There is a shift toward developing more personalized risk profiles and using decision tree techniques that explore hundreds of predictors rather than a few clinically relevant risk factors [31]. Modern machine learning techniques are better placed to identify complex relationships between large datasets and suicide risk [13].

Early evidence generated by a pilot study using data from 144 patients with mood disorders suggests that machine learning algorithms using previous clinical data were successful in distinguishing between people that attempt suicide and those who do not, with a prediction accuracy between 65% and 72% [32].

Although there has been a growing body of research seeking to augment or advance traditional methods with the aid of machine learning in clinical psychiatry [2,4,29,30,33-37], the majority of studies rely on applying algorithms that learn from clinical data such as health care and electronic medical records, unstructured notes by providers and caretakers, or some other data carefully gathered by health care professionals.

### Objectives

In this feasibility study, we aim to add to existing knowledge by using a nascent approach combining clinical data with proxy risk active and passive data collected from mobile devices to develop our algorithm. We developed a software platform to collect data on inpatients in acute mental health settings via our own mobile app, *Strength Within Me* (SWiM); a smartphone (iPhone); a wrist wearable (Fitbit; Fitbit, Inc); and questionnaires administered by the research team. Active risk data—patient-facing user interface modules (eg, journaling, safety plan, and mood meter)—and passive risk data that did not require direct interaction from the patient (eg, sleep monitoring) were collected behind the scenes. This information was then used to construct and train machine learning algorithms seeking to produce a risk score that deduces the likelihood of suicide. We used the risk level from the Columbia-suicide severity rating scale (C-SSRS) [38], which was assessed by mental health researchers as our standard classification target. C-SSRS is currently considered the *gold standard* approach for the measurement of suicidal ideation and behavior in clinical trials [39]. Previous research has confirmed the validity of the scale and its prediction accuracy for short-term risk of suicidal behavior in clinical and research settings. Studies have demonstrated that individuals who meet the criteria of high risk following the administration of C-SSRS are almost 4 times more likely to attempt suicide within 24 months [38]. The C-SSRS was then compared with data from proxies for risk factors [40] such as sleep quality and emotional health collected via Fitbit () trackers and the SWiM app that patients interacted with for a week during their admission.

## Methods

### Participants and Clinical Setting

**Figure 1:**
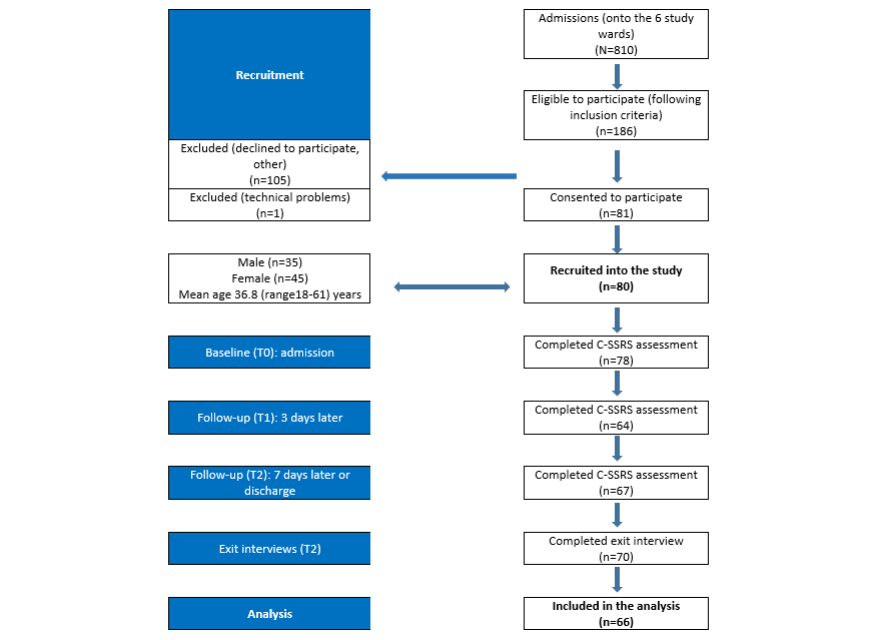
Strength Within Me study flow diagram. Timeframe for recruitment: January-November 2018. Included in the analysis: participants who completed C-SSRS at second follow-up. C-SSRS: Columbia Suicide Severity Rating Scale.

In this phase 1 feasibility study, we collected data from service users admitted to 6 acute adult mental health wards within a National Health Service trust in the North West of England, United Kingdom. Service users who had been admitted to a ward within the last 7-10 days were assessed by nursing staff to determine study eligibility. Following informed consent, participants were given a study iPhone and Fitbit to enable use of the SWiM app and monitor their sleep and daily activity for up to 7 days. Participants were then involved in 3 interviews at three different time points (ie, as soon as possible following admission, 3 days later and 7 days later or at discharge, whatever came first) to complete a battery of assessments, including the C-SSRS [38], examining suicidal thoughts and behavior. The interviews were completed by 2 experienced researchers who were trained to administer the clinical assessments. If suicidal risk was highlighted during the interview, nursing staff were informed and an agreed protocol was followed to ensure safety. Participants were given vouchers following the completion of assessments. In total, 80 patients out of the 186 eligible consented to participate and 66 were included in the analysis based on the completion of at least two follow-up clinical assessments. This represents a 43% response rate and 83% completion rate. For a breakdown of participants, see Figure 1*.*

The authors assert that all procedures contributing to this work comply with the ethical standards of the relevant national and institutional committees on human experimentation and with the Helsinki Declaration of 1975, as revised in 2008. All procedures involving human subjects, ie, patients were approved by the Health Research Authority in England and the North West–Liverpool East Research Ethics Committee (Reference 17/NW/0173). Written informed consent was obtained from all inpatients.

### Overview of Participant Data Fed Into the Modeling Process

At a high level, the data are segmented into (1) data entered by the user into the SWiM app; (2) data collected passively by the SWiM app, the Fitbit wearable, and the Apple Health app; (3) data directly gathered by our researchers; and (4) social interaction data for those who gave permission. We gathered a total of 173 variables—a mix of raw data such as counts of the number of journal entries and derived values that involve summary statistics or other variations of the data (eg, adding up minute-wise sleep records to get total sleep time or the number of interruptions in sleep). Social interaction data were excluded from the analysis because of the low response rate, that is, 8 out of the 80 participants gave permission or had access to Facebook.

As shown in Table 1, user-inputted data included participant mood, free-form journal entries, steps for personal safety plans, and custom reminders they could have set for themselves. From these entries, we collected descriptive statistics such as average mood reported (Likert Scale 1-5), average character limit, maxima, minima, and raw counts. A particular derived variable of interest from journaling was the average sentiment derived for each journal entry. This sentiment (ranging from −1 for negative to +1 for positive) was calculated via a third-party model known as the Valence Aware Dictionary and Sentiment Reasoner [41], which is catered to sentiments expressed in social media but has proven itself in other domains. The idea behind using this model was to obtain a proxy for the indication of feelings by users as they write and reflect.

Data collected by the research team included sociodemographic information, such as age and gender, and clinical assessment data. The key information that we used in the modeling was the researchers’ assessment of the patient through the C-SSRS, which was assessed a maximum of 3 times (patient entry, 1 or two follow-ups during their hospital stay, and exit). All 80 consenting users were at risk upon entry to the ward (when the first test was done), so at this point, no prediction was done. The initial thought was to compare results against an intermediate survey result and exit survey result, considering the change in risk, but we did not have enough exit surveys for 2 different time period comparisons. Overall, 66 out of the 80 participants had taken at least a second survey where risk level was reassessed, and that was the population included for prediction. There was a 3-7 day wait from the first assessment to the second assessment.

Finally, we included passive data gathered via the phone and the Fitbit wearable, such as details about a user’s step frequency and count from Apple’s Health kit app, minute-level sleep data from Fitbit, and engagement patterns with the phone (eg, number of log-ins to the SWiM and the number of times a certain section was visited). Levels of engagement with study data are presented in Table 2.

**Table 1:** Strength Within Me study data.

Data source	Examples of variables collected	Examples of raw data	Examples of derived data
Facebook	Stats of Facebook activity and post activity	Number of posts: 5 and number of total likes: 100	Average likes per post: 20
User input	Journal, mood, reminders, and safety plan steps	Journal entry: “Last night was horrible. I couldn’t sleep at all with the noise.”	Sentiment: −0.8 and word count: 12
Clinical team	Demographics and C-SSRS^a^ responses	Age: 35 years and C-SSRS risk overall: moderate	C-SSRS risk binary: 1
Passive sensor data	Sleep, steps, and interactions	{*dateTime*: *23:10:00*, *value*: *awake*}, {*dateTime*: *23:11:00*, *value*: *asleep*}	Sleep latency: 1 min and average time asleep: 5 hours

^a^C-SSRS: Columbia-suicide severity rating scale.

**Table 2:** Engagement rate across active and passive data in the study (N=66).

Data source	Rate, n (%)
Step-related features (Fitbit and iPhone)	26 (40)
Journal entries (self-documented via SWiM^a^ app)	45 (68)
Mood entries (self-reported via SWiM app)	53 (80)
Phone activity (data usage)	66 (100)
Sleep (Fitbit)	59 (90)

^a^SWiM: Strength Within Me.

### Modeling

#### Machine Learning Setup and Data Analysis in Our Clinical Setting

As a first step toward developing an algorithmic risk score that is valid in predicting suicide risk, we framed the problem as a supervised, a binary classification problem in which users were categorized in terms of levels of risk of *low risk* versus *high risk* using the information specified above. These *low risk* and *high risk* labels were derived from the overall C-SSRS risk scores obtained after asking participants a range of questions on previous attempts, ideation, etc. Usually the 3 categories are *low*, *moderate*, and *high*, but we grouped *moderate* and *high* for the sake of tractability from a modeling perspective. From a machine learning perspective, this aids in what is commonly referred to as the *class imbalance* problem [42], where certain categories have relatively few labels to their other counterparts. This makes it statistically more difficult to identify, and these categories as models are inclined to achieve high scores by predicting the most common class; we turned a distribution of 36 low, 5 moderate, and 25 high to 36 low and 30 high. Choosing a binary case was helpful in dealing with the class imbalance issue, as models are data-dependent in terms of volume (ie, the more examples, the better job they do in learning). This is especially critical when we take into account the limitations in our data; to fairly judge the model performance, we must partition the data (a test set and training set via k-fold cross-validation [43]) to assess how well the model can predict risk on *new users* given what it is learned from *old users* [44]. From a risk-app perspective, although it would be ideal to place users on a continuum of risk levels, it is critical to first assess the feasibility of identifying users at discrete thresholds as well as seeing the degree to which we can match the current standard in risk assessment.

Our *low risk* and *high risk* categories were mapped to binary outputs of 0 or 1. Some features derived from a user’s journal entry are the word length and sentiment score (ranging from negative with −1 to positive +1; for further information, please refer to the source model from which this is derived [41]). To account for the time dependency in the data (multiple journal entries across multiple days for example), a majority of the features engineered were done so in a *summary statistics* fashion (mean, median, variance, etc). For example, the average journal word count per day over the user’s total number of entries was used to summarize one aspect of a user’s journaling behavior over their time with the app.

We curated 172 features formulated from categories of sleep data, journal entries, data usage, mood, and app activity statistics. For more information, a comma-separated values file including the full list of features incorporated into modeling (besides *uid*, which is user id to anonymize yet identify patient) is included in Multimedia Appendix 1. The 172 features were projected down to a 5-dimensional space by principal component analysis (PCA). This sample provides an insight into replicability. Any feature that has a summary statistic attached such as *mean* or *std* was done over the course of the 3-5 days before the second assessment. Categorical features such as gender were mapped to numerical (in this case binary) outputs for the algorithm to consume.

This is typically considered a relatively high number of features relative to the amount of possible supporting data points per number of users recorded. To provide a more suitable set from which a machine learning algorithm may distinguish a signal for risk, we turned to feature selection and dimensionality reduction techniques. Our aim was to cut down to a smaller set of features that may also be interpretable and grounded in clinical knowledge of risk factors. We, therefore, opted for PCA [45] as our dimension reduction technique and used Random Forest [46-48] to help in terms of feature selection as well to check the reliability of our reduction. Algorithms such as SVMs [49] are designed in such a manner as to overcome dimensionality issues, but they were experimentally confirmed to be unsuited to the task due to the size of the data.

**Figure 2:**
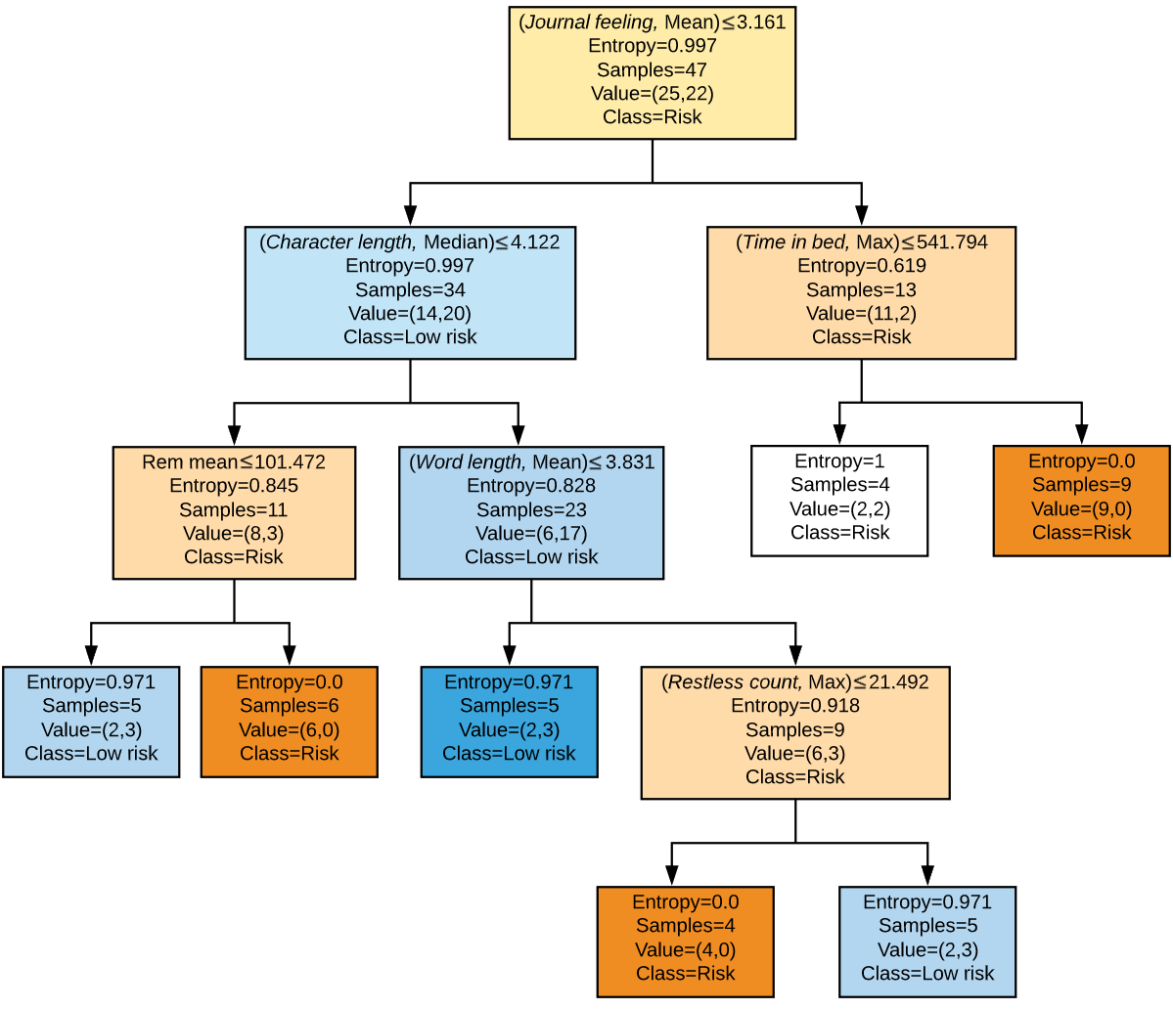
Example of a decision tree formed for the Strength Within Me study data.

For our study, the random forest model was composed of 25 decision trees. We took a look at the top 30 of the 170 original features and found that journal-related features such as average feeling, cell activity such as the variation in user’s data usage, sleep-related features such as average sleep efficiency (time spent sleeping or total time spent in bed), and other natural indicators, mostly known to clinical psychology, are markers of risk. For an example of a decision tree formed for our data, see Figure 2*.* The tree is read similarly to a flow chart in a top-down, left-right fashion. For example, at the top, we start with an entropy of 0.997 (entropy of 1 means complete uncertainty with 0 as certainty) [19-21], as we have 25 people in the low-risk category and 22 in the high-risk category. We, then, look at their average journal feeling, and if it is less than 3.161, we go to the left node with a subgroup of 34 people, otherwise the right node with a subgroup of 13 people. Following the right node, we now have a subgroup of 13 people with an average journal feeling greater than 3.161. On the basis of this characteristic alone, we reduce entropy to 0.619 (we are more certain of our group) and have 11 users correctly identified as high-risk, but 2 low-risk users misclassified as high-risk users. Reduce misclassification: we again split by the average amount of time the user has spent in bed. If they have greater than 541 min spent in bed in a day, a subgroup of 9 out of 9 people is correctly identified as high-risk users. However, we see that for less than 9 hours, we also predict high risk and have complete uncertainty (entropy 1), as the subgroup of 4 people is evenly divided among the classes. Once we reach 1 of these leaves or terminal nodes, we can read the decision process used to get there. For example, for the right-most leaf with 9 samples we discussed, users with an average journal feeling greater than 3.161, who also spend more than 9 hours in bed, are identified as high-risk users. Similar interpretations can be made for the other 6 terminal nodes. Worth noting is that the features are ordered top-bottom in terms of ability to split classes and reduce entropy; by this criterion, we see journal feeling as the *most important* feature, time in bed as second, and so on.

#### Principal Component Analysis

**Figure 3:**
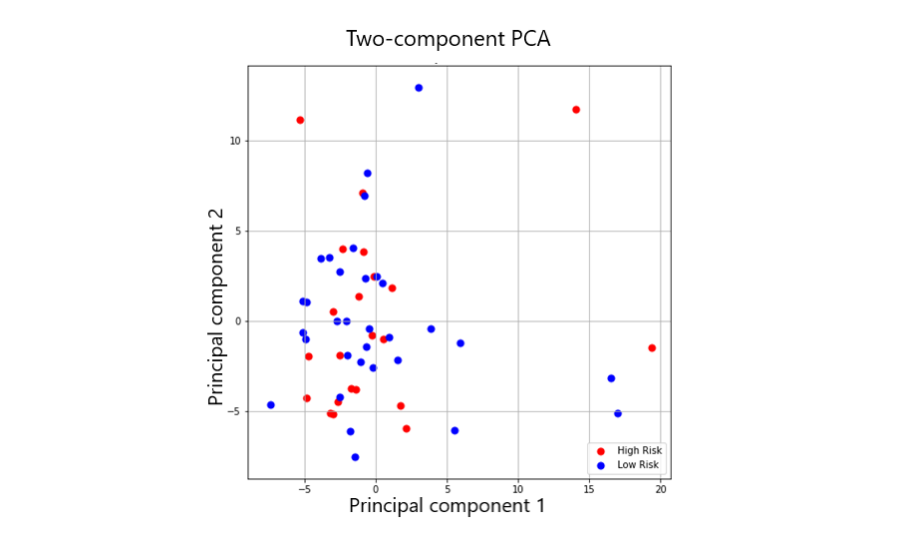
A diagram of principal component analysis. A high-dimensional dataset has been flattened to a 2-dimensional space where the new axes correspond to the principal components (they point in the direction of the largest variance of the data).

For dimensionality reduction and to guard against overfitting, we turned to PCA. On a high level, PCA groups features that are correlated with one another into new features (principal components) that hold the most signal in terms of variation in the data [44]. The idea is that features that explain a high level of variability found in the data produce most of the signal needed to distinguish categories. Those features which do not contribute as too strongly are discarded; by doing so, we can obtain a concise set of features at the cost of a small drop in prediction performance. Formally, PCA is an orthogonal linear transformation that maps the data to a new coordinate system such that the bulk of the variance of the projection is covered by the first k components, where k< total number of original features and components are linear combinations of the originals. Another important characteristic of PCA is that it is not optimized for class separability and may be considered as an unsupervised model. This is critical as we aim to achieve generalizations outside of the data at hand, and we do not want to overfit our final model. To provide a visualization of the PCA transformation on our data, an example of a 2-dimensional, 2 components PCA is given in Figure 3*.*

After looking at the variance captured up to 100 possible components, we settled for the first 5 components, as they accounted for 55% of the variance. Our first 5 components are described in Table 3, along with the themes/patterns identified after reviewing which features were grouped. We were assured that these components made sense in terms of clinical knowledge of how sleep quality, mood, activeness, and other characteristics are indicators of mental health [50-53]. Moreover, the top 30 features of our feature selection from random forest strongly overlapped with these features, and so we were further assured in terms of potential predictive power.

**Table 3:** Principal components analysis components and patterns.

Component	Description	Themes, patterns
First component	Maximum efficiency, average efficiency, median efficiency, max time in bed, and number of sleep recordings	Ability to sleep, sleep quality
Second component	Number of packets sent, number of times connected to Wi-Fi, number of times connected to cellular data plan, and number of times journal entered	User app activity, data presence
Third component	SD sleep start, median journal feeling, max sleep start, max journal feeling, minutes in bed, and minimum journal feeling	Feeling versus sleep activity
Fourth component	Median char length, median word length, median journal feeling, SD rest duration, and max rest duration	Journal input versus resting variability
Fifth component	Median sentiment, SD number of awakenings during sleep, number of awakenings during sleep, and minimum sentiment	Sleep quality and reflection tone

## Results

### Prediction Algorithm Testing

We tested a series of algorithms that we thought would be best suited to predicting levels of risk from a theoretical perspective. Often referred to as the *bias-variance trade-off* [44,54,55], there is often the case with model selection that the best model should not be too simplistic such that its crude predictions miss a bulk of the cases, nor should it be overly complex such that its high sensitivity perfectly fits the data, but fails to generalize to new, unseen data. This principle, along with other individual algorithm properties, helped guide the experimentation. As discussed in the literature [56], increasing the complexity and flexibility of a model tends to allow it to understand more nuanced relations but at the cost of being overly sensitive to noise within data and overfitting. Hence, not only were models of varying complexities chosen for comparison from linear models such as logistic regression to nonparametric models such as K-nearest neighbors (KNN), but the parameters within each model were also tuned by choosing the number of neighbors and reducing dimensionality through PCA.

It is important to mention that these models are selected and judged based on various metrics that aim to capture the objective for which the model is needed. Certain metrics also have advantages over others depending on the imbalance of classes, nature of the data (categorical or numerical), and other factors. As we had a nearly balanced dataset, and this was a feasibility study, we opted for the simplest way to measure performance, in this case accuracy, to understand metric of accuracy where we measured the number of correctly predicted observations over the total number. As a baseline, we looked at the simplest heuristic of predicting the majority class of low-risk users. This produced an average accuracy of 53%.

Random forest was tested as it is generally agreed upon as a strong *out-of-the-box* model that performs well on various datasets in different contexts as well as having interpretability through the feature importance it can help provide [46,48]. Logistic regression was another model considered due to the log-odds interpretability for the coefficients to each of the features (usually referred to as explanatory variables in explanatory contexts) and natural fit to classification problems [44]. SVMs [44,49] were also tested as they have the design of naturally combating the *curse of dimensionality* through the transformations they do to the data (*kernel trick*). SVMs are also rather sophisticated models that tend to produce near state-of-the-art results (barring neural networks which at the time of writing are highly data-hungry, and not necessarily interpretable). Finally, we considered the KNNs algorithm, which is often sought due to simplicity as well as the natural heuristic of classifying based on how *close* observations are to one another [57].

To test the performance, we performed k-fold cross-validation with k=10. This means that we randomly partitioned the data into 10 pieces (folds) and used 9 of them to train the model and 1 as an *unseen* piece (fold) to test on. This was done such that each of the 10 folds was used as the *unseen*/testing data at a given iteration. The idea was to obtain the expected accuracy of a model when exposed to new data by simulating variations of data seen to unseen data. We repeated this process 10,000 times to obtain a more stable estimate, as there are many ways to partition this data into 10 folds. Table 4 summarizes the results.

**Table 4:** The average cross-validation accuracy, along with the SD of the accuracy observed for the various folds.

Algorithm	10-fold CV^a^ average accuracy (10,000 iterations)	SD	Comments
K-nearest neighbors (k=2)+PCA^b^ (n=5)	0.68	0.12	Best performance, k=2 seemed natural and worked best up to 10
Random forest (k=25)+PCA (n=5)	0.60	0.13	Nonlinear helps, too many trees did not, PCA reduced deviation
Random forest on raw features (k=25)	0.60	0.15	Nonlinear helps, too many trees did not
SVM^c^ (degree 2 polynomial kernel)	0.57	0.10	Likely overfit, base guessing
Logistic regression+PCA (n=5)	0.59	0.14	Removed correlation due to PCA+prevent overfitting
Logistic regression on raw features	0.55	0.16	Likely overfit, base guessing
Baseline: guessing majority from training fold	0.53	0.20	Baseline to beat

^a^CV: cross-validation.

^b^PCA: principal component analysis.

^c^SVM: support vector machine.

Logistic regression failed to perform much better than baseline. With the raw features, it performed poorly likely due to overfitting and high collinearity between some features (eg, median sleep time and mean sleep time). We removed most of this through PCA and performed slightly better on average at 59%, but the SD of 14% was worrying, given its below baseline lower end (worse than majority guessing). Similarly, SVM failed to perform much better, and of the different kernels, we present the polynomial degree 2 kernel as it performs best out of other variations (higher order polynomials, radial basis kernels, and linear). We defer the explanations of these kernels to the literature. Random forest performed better than either of the other 2 algorithms, but worst-case folds still fell below baseline.

### The k-Nearest Neighbors Algorithm

**Figure 4:**
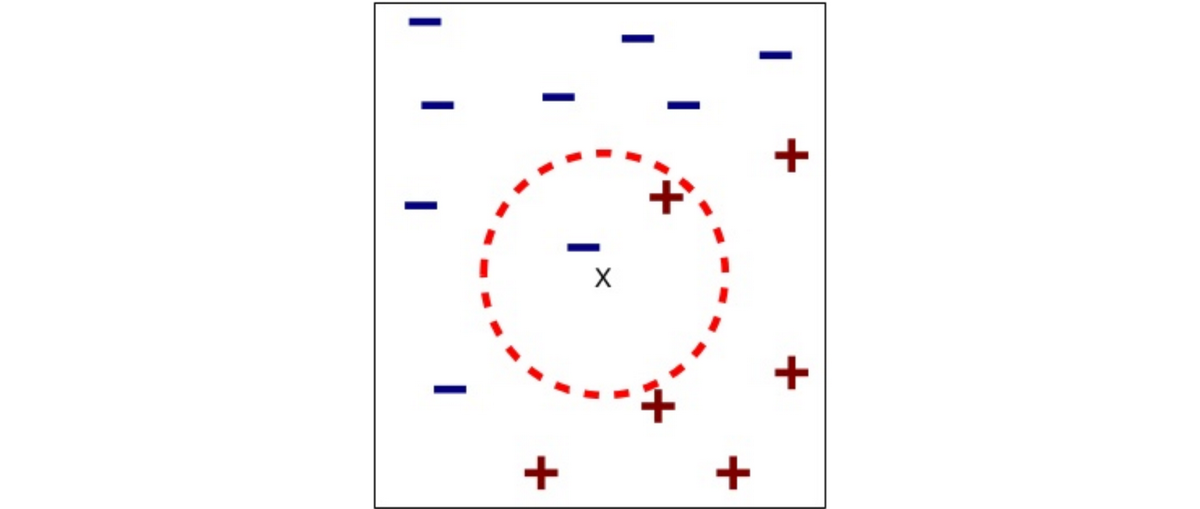
Example of nearest neighbors with k=2 with data in 2 dimensions. Here, the new test point is x and has 1 minus neighbor and 1 plus neighbor as its 2 closest neighbors. As the minus neighbor is closer, the new point x will be classified as minus. “+” stands for positive class, “-” for negative class, and “x” for new data point that has yet to be assigned a class.

**Figure 5:**
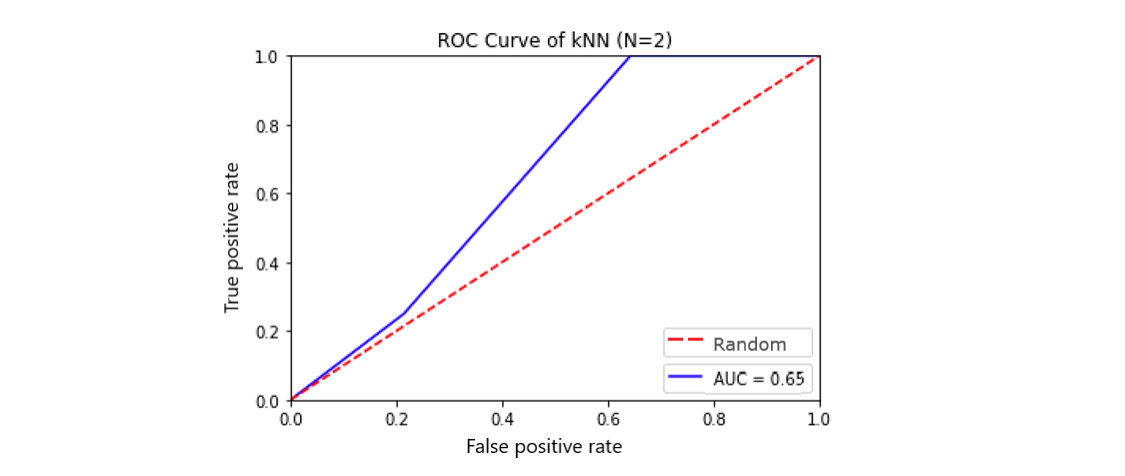
Receiver operating characteristic curve for k-nearest neighbors. AUC: area under the curve.

The most promising was the KNNs algorithm with k=2 with the 5 principal components discussed earlier as features with not only an average accuracy of 68% (averaged over 10,000 simulations of splitting the training and testing data via 10-fold cross-validation) but with an SD of 12%, which resulted in its worst performance just above baseline at 56% and upper limit of 82% (Figure 4). In terms of false positive and true positive rates, the model achieved an average AUC of 0.65 (Figure 5). We applied a 5×2 cv combined *F* test to test the model performance of KNN against the baseline classifier that guesses training majority, random forest, and other models such as supported vector machine and logistic regression and achieved *F* statistics of 10.7 (*P*=.009) and 17.6 (*P*=.003) for training majority and random forest, respectively, rejecting the null performance being the same [58,59].Due to the promising results of the algorithm, we explain it to readers unfamiliar with it. The KNN algorithm essentially follows the saying of *birds of the same feather flocking together*. That is to say, the way prediction is performed using this algorithm is that for a new test point, the distance (usually the well-known Euclidean distance) is computed between the new point and k of the closest previously labeled observations. Of the k neighbors, the majority class is chosen as the label for the test point. For example, with k=5, we look at the features of a new person whose risk has not been identified yet and look at 5 people with the features closest matching this new person out of the training set. If 3 of them are high risk and 2 are low risk, the new person is identified as high risk, with 3 votes to 2. For even numbers of k, such as 6, where there might be ties, we weight the votes by proximity. Therefore, with respect to our PCA features, we are comparing people who have similar sleep characteristics, data usage, and so on. We found k=2 to perform best in our scenario, likely due to the low sample size as well as high variability among users. We used a Euclidean distance metric and enforced each feature to have equal distance weighting (uniform weights).

## Discussion

### Principal Findings

The results from this feasibility study indicate that, although not a perfect predictor, the KNN model is suitable for this study because it has shown the ability to separate users deemed at risk of suicide from the C-SSRS to those not deemed at risk at an average rate beyond just randomly guessing (ie, at an average rate 15% beyond randomly guessing the majority to be at low risk). These are early indications that it is possible to predict risk using the data collected in this feasibility study, using the KNN algorithm. The data used to inform this included users’ sleeping activity, step activity, self-reported mood, journaling thoughts, and activity levels as measured by a phone app.

This is a crucial first step in automatic risk assessment, as we managed to build an algorithm that predicts suicidal risk at a rate significantly better than the baseline of guessing the simple majority that were collected directly from smartphone interactions. This is also promising, as we are working with a relatively small dataset from a machine learning perspective. This is the basis for future phases of this study, where we will be looking to test the model on additional users of mental health services for further testing of concept and generalizability.

The implications of this feasibility study are highly significant for building capacity for suicide risk prediction (future risk) or detection (real-time, current risk). With a low proportion of suicide attempters who actually access mental health services [60], it is essential to develop and test nonclinical means of assessing risk. Given the dynamic nature of suicide ideation and suicide risk, new methods are needed to track suicide risk in real time [61], together with a better understanding of the ways in which people communicate or express their suicidality [25]. Mobile apps could be better suited to help prevent suicide by offering support in situ and at the time of crisis [62].

Although previous studies have utilized electronic health care record data to create an actuarial model of suicide risk [30,34,35,37,63-65] or focused on a single aspect of user input such as language [29,30,36], this study adds to the literature by introducing external, user-generated input, and smartphone data and combining it with clinical data. Our study adds to evidence that reports on the use of external, nonclinical data to predict suicidality. The results are promising, although we used basic, simpler, and routine biometrics (collected via iPhone and Fitbit), compared with data used in previous research. Studies aiming to predict mental state (short term) have used multiple (self-report) measurements and a wide range of bio sensors [12,15,66].

### Strengths, Limitations, and Further Testing

We recognize that our study is limited by the short follow-up period of up to a week; thus, future iterations would need to extend to a longer period of study to explore the time sensitivity of model predictions over varying time windows (eg, predicting current risk vs 1 week out). Short-term risk prediction is difficult because any inference is based on limited data, which means that meaningful signals are lost due to noise from highly variable behaviors [13]. There is promise in improvement as the amount of data available for training and testing increases. Previous research and machine learning literature [67-70] points to expected improvement in performance and reliability in test results as the sample size grows, particularly in this classification setting. We expect that roughly doubling the sample size would achieve more practical results where the possibility of implementation would be appropriate.

Although the results from this feasibility study have been promising in producing a signal, in terms of operationalizing risk for suicide, future steps would be moving beyond survey-generated risk scores. Before taking that leap, the intermediate step would be to further validate the algorithmic results by collecting additional, more substantial test data. Where the experiment excels in the data sources are diverse rather than strictly clinical and allow for natural extension to outpatient settings. In addition, given the probabilistic nature of the algorithm, there will naturally continue to be a trade-off between false positives and false negatives as the model improves, and hence, medical, human attention in decision making will remain critical. We propose that the algorithmic approach provides a supplement and an additional facet to clinical judgment.

Therefore, having achieved a signal from the data for risk in phase 1, phase 2 (proof of concept) will involve collecting more data to not only see if modeling improves but also to test other models such as predicting the risk score trajectory. Enforcing a minimum of 2 C-SSRS assessments, we can try to model changes in risk. We also intend to experiment with more features, particularly those involved in text mining, as most journaling features were relatively surface level. Moreover, we aim to look at prediction stability over time, as this prediction was made within a couple of days from usage to assessment.

Our final aim is to form our own standard so as to break away from dependency on the C-SSRS, as we look to go beyond information gathered in a formal survey that depends solely on human judgment. Further research will enable us to test the viability of automation and machine learning to identify suicide risk by comparing predictions of risk with eventual outcomes as well as testing out the model in different settings and populations (eg, community).

We would also like to point out that, although mobile phones and apps are ubiquitous and have the potential to be an efficient and cost-effective approach to addressing mental health problems [71], this study indicated that there are certain costs that limit the widespread adoption of health apps within mental health services (weather inpatient and community settings). These are related to access to smartphones, connectivity, updating, and maintenance of technology. The premise for this study was that, in line with the UK population statistics indicating that approximately 95% of households own a mobile phone [72], of which a high proportion are smartphones, participants would have access to and use their own smartphones for the study. Following initial scoping, the authors realized that only a small proportion of inpatients had access to a smartphone. In addition, the SWiM app was configured (in its current testing form) to operate only with iOS products, that is, an iPhone. We cannot confirm the extent to which given participants study iPhones might have affected the results; this is something that needs to be further explored. We can, however, highlight that participants were enthusiastic about using Fitbit wearables and the Fitbit app on the phone, which may or may not have encouraged them to use the SWiM app as well.

### Conclusions

Although in its early stages, research in this area suggests that using smartphones to enquire about suicidal behaviors can be a valuable approach and not a risk factor for increasing suicidal ideation [12]. Given the heavy reliance on smartphones and mobile devices around the world, this readily available source of data is an important and highly underutilized source that has good potential to improve mental health risk prediction and prevention and advance global mental health.

However, although full automation and independence of clinical judgment or input would be a worthy development for those individuals who are less likely to access specialist mental health services, and for providing a timely response in a crisis situation, we need to acknowledge the ethical and legal implications of such advances in the field of psychiatry [72,73]. The use of machine learning in suicide prediction needs a strong evidence base across different settings, populations, suicidal behaviors, and datasets, before considering full integration in health care settings. For the time being, if proven accurate and scalable, machine learning algorithms for suicide risk detection are likely to complement rather than replace clinical judgment [72]. Although smartphones provide us with opportunities to gather data on real-time dynamic risk factors for suicidal behavior, which would be almost impossible to monitor on discharge (from mental health settings), more research is needed to validate the utility of risk markers for suicide behavior and confirm a safe and clinically effective way to use these data to inform practice [13]. More work is needed before we can achieve safe and effective integration within mental health settings, while remaining attentive to key ethical implications. An interesting ethical dimension is related to the use of the KNN algorithm, which requires continued access to the pooled data of (at least a subset of) multiple participants to subsequently label new cases. Although testing this in a controlled setting with inpatients who have provided consent for the use of their data might be straightforward, it is uncertain if service users in the community would accept to have their suicidal trajectory data shared for this purpose or how mental health services would be able to bridge the gap. Furthermore, to achieve high accuracy in terms of short-term risk prediction, a wide variety of data from multiple sources will need to be collected, with data integration as a key component [13]. We, therefore, expect multiple data governance, privacy, and intellectual property issues at stake.

## Appendix

Multimedia Appendix 1 Sample training data.

## Abbreviations

C-SSRS: Columbia-suicide severity rating scale
SVM: support vector machine
SWiM: Strength Within Me
